# Physically Transient Gelatin-Based Memristors of Buildable Logic Gates

**DOI:** 10.3390/gels11060428

**Published:** 2025-06-03

**Authors:** Lu Wang, Yuting Wang, Wenhao Li, Zhiqiang Gao, Yutong Han, Dianzhong Wen

**Affiliations:** School of Electronic Engineering, Heilongjiang University, Harbin 150080, China

**Keywords:** gelatin, Au NPs, physical transient, NAND, NOR

## Abstract

Moore’s Law is being challenged, as the use of transistors has limitations in terms of physical materials, energy consumption, performance, and economics. To continue Moore’s Law, people have put forward many ideas, one of which is to find smaller devices to replace CMOS transistors. Memristor-based digital logic circuits open new avenues for exploring advanced computing architectures. In this paper, a biomemristor with the structure of Al/gelatin:Au NPs/Al/gelatin was fabricated using gelatin as the substrate and the host material of the dielectric layer. The device has a large switching current ratio, good stability, and physical transient characteristics. The device can be dissolved by soaking in deionized water for 5 min. In addition, the device successfully realizes the functions of NAND and NOR logic gates. It provides an effective method for research on green electronic devices with logic functions.

## 1. Introduction

With the explosive growth of digital transmission in the age of artificial intelligence and big data, artificial intelligence technology has made great strides [1,2,3,4,5]. Due to the physical separation of memory and processor in the traditional von Neumann architecture, computer systems with huge memory capacity and excellent processing efficiency urgently need to be developed in various fields, such as machine learning and network monitoring [6,7,8,9,10]. However, continuous scaling down to increase the number of devices per unit area and thus increase storage capacity also has limitations due to quantum uncertainties and fabrication complexity. On the other hand, the frequent access and transmission of data between the central processing unit and the memory greatly increases energy consumption, thereby reducing information processing efficiency. Therefore, challenges and obstacles in Moore’s Law and traditional von Neumann computing architectures need to be considered [11,12,13,14,15,16,17]. Among various resistive random access memories, resistive random access memory (RRAM) has been widely studied due to its advantages of convenient access through interconnected electrodes, low energy dissipation, strong scalability, and strong integration capability [18,19,20,21,22,23,24,25,26].

In recent years, memristors have emerged as promising candidates for next-generation nonvolatile devices due to their potential in digital logic and unconventional computing architectures. Constructing logic gates using memristors offers a new pathway to overcome the scaling and energy challenges faced by traditional CMOS circuits. Within this context, researchers have increasingly explored bio-memristors based on natural materials, aiming to achieve environmentally friendly and sustainable device platforms capable of logic and synaptic functions. Several bio-memristor structures have been reported. For instance, a device based on tussah hemolymph has been demonstrated to implement AND and OR logic operations, with a switching ratio of ~10^2^ and a retention time of 10⁴ seconds [27]. Another study developed a bio-memristor using a mugwort-PVDF composite layer, achieving multiple logic gate functionalities (AND, OR, NAND, NOR, XOR), although its switching ratio was relatively low (~3.6) despite excellent retention (~10⁵ s) [28]. A silk nanofibril-based device has also been shown to realize basic logic functions while maintaining mechanical flexibility [29]. In addition to logic operations, some bio-memristors have demonstrated neuromorphic behaviors. For example, a device based on silk protein exhibits short-term plasticity and paired-pulse facilitation, mimicking synaptic response behaviors [30]. Another sericin-based device enables the modulation of synaptic strength through spike-rate- and spike-timing-dependent plasticity [31]. Overall, although previous studies have demonstrated either logic or synaptic functions based on biomaterials, most devices still face trade-offs in terms of electrical performance, logic complexity, and degradability. Therefore, it remains a significant challenge to develop bio-memristors that integrate high electrical performance with logic functionality and environmental compatibility.

To construct physically transient memristive devices with logic functions, we selected natural gelatin as the active layer material. Gelatin is widely available, low-cost, and exhibits excellent flexibility, biocompatibility, and biodegradability. Its abundant polar functional groups facilitate stable ion migration and charge transport. Meanwhile, the embedded Au nanoparticles (Au NPs), with their nanoscale dimensions, induce a Coulomb blockade effect, which suppresses continuous charge transport and enhances the resistance in the high-resistance state, thereby enlarging the switching current ratio. The resulting device not only successfully realizes NAND and NOR logic gate operations but also demonstrates a high switching current ratio (~7.68 × 10⁶) and a stable retention time (10⁴ s). Furthermore, the device is fully water-soluble and can be completely dissolved in deionized water within 5 min, indicating strong physical transient characteristics. As shown in Table 1, we systematically compared our device with other recently reported bio-memristors. The results reveal that our device offers more comprehensive advantages in terms of electrical performance, logic capability, and biodegradability, providing a valuable reference for the development of green logic electronics.

## 2. Results

**Figure 1:**
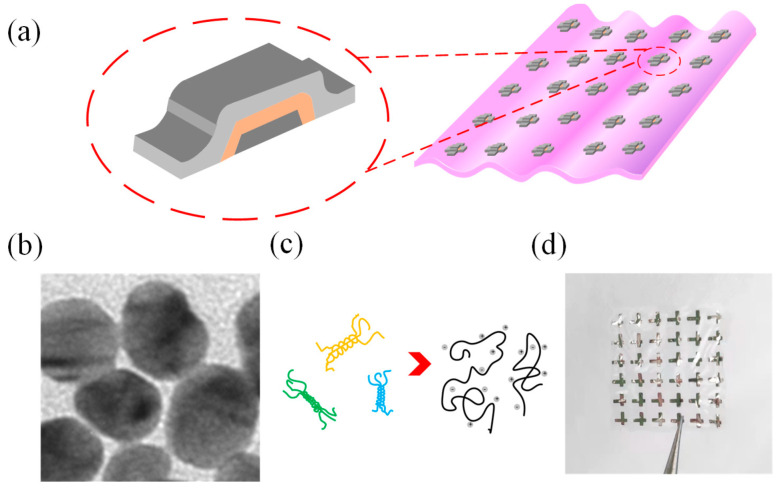
(**a**) Schematic diagram of the structure of the Al/gelatin:Au NPs/Al/gelatin biomemristor. (**b**) TEM image of Au NPs. (**c**) α-helical structure of gelatin chains. (**d**) Photograph of the Al/gelatin:Au NPs/Al/gelatin flexible biomemristor.

Gelatin is made from animal collagen by heating and hydrolysis. The structure of gelatin is Ala-Gly-Pro-Arg-Gly-Glu-4Hyp-Gly-Pro [32]. It has a heteroatom (i.e., nitrogen or silicon) that is strongly coordinated with the metal ion and has a conductivity that allows current to flow. Figure 1a is a schematic structural diagram of the Al/gelatin:Au NP/Al biomemristor, in which Al is the top and bottom electrodes, the gelatin:Au NP composite film is the medium layer of the device, and the transparent flexible gelatin is the substrate. Figure 1b is the TEM image of Au NPs, and the diameter of Au NPs is approximately 10~20 nm. Figure 1c is an alpha-helical structure of gelatin chains. Figure 1d is a photograph of the device, from which it can be seen that the gelatin substrate is transparent. The cross-sectional transmission electron microscopy (TEM) image of the Al/gelatin:Au NPs/Al device structure is provided in the Supporting Information (Figure S3), confirming the layered structure and corresponding thicknesses.

**Figure 2:**
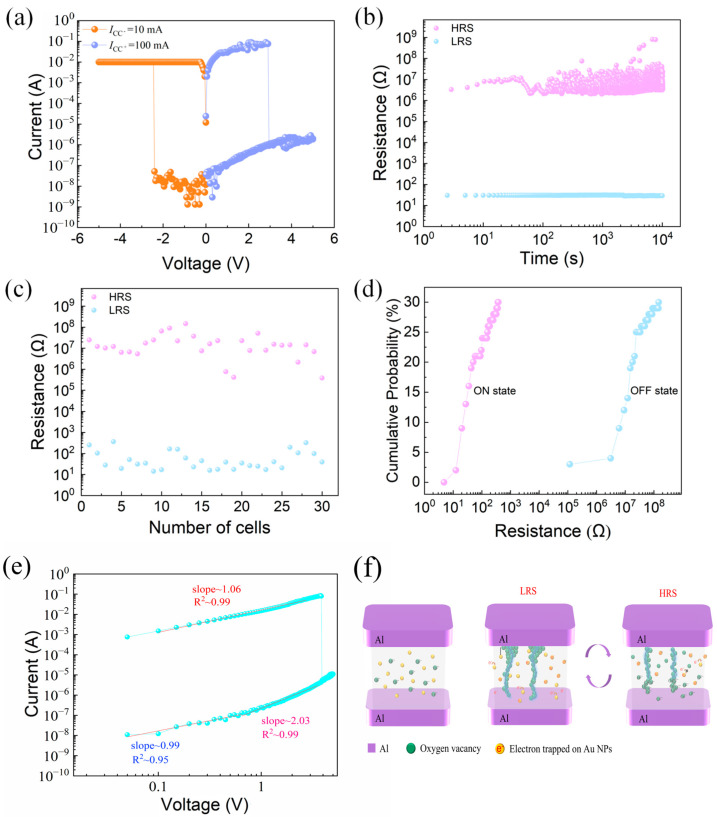
Al/gelatin:Au NPs/Al/gelatin devices. (**a**) Typical current-voltage (I–V) curves. (**b**) Hold time. (**c**) Resistance values of the HRS and LRS for 30 memory cells. (**d**) Resistance cumulative probability. (**e**) Double logarithmic plot of the I–V curve under positive bias, showing three conduction regions fitted with Ohmic and SCLC mechanisms. (**f**) Schematic illustration of the resistive switching mechanism, including formation and rupture of oxygen vacancy conductive filaments.

Figure 2a shows the typical I–V characteristics of the device, demonstrating clear resistive switching behavior. We highlight that no forming process was required for the devices in this study. The resistive switching behavior was directly observed upon applying the measurement voltage, which eliminates the complexity of an initial forming step. This characteristic enhances the simplicity and reliability of the device operation. The limiting current I_CC−_ = 10 mA is set under negative bias, and the limiting current I_CC+_ = 100 mA is set under positive bias. Continuous voltage sweeps are performed on the device. The maximum switching current ratio is 7.68 × 10^6^. The retention characteristics of the LRS and HRS of the device tested under constant voltage are shown in Figure 2b, and the LRS and HRS of the device did not fluctuate significantly within 10^4^ s. I_CC−_ = 10 mA and I_CC+_ = 100 mA were set, and the electrical characteristics of 30 memory cells of the same device were tested. The current values of the HRS and LRS are shown in Figure 2c, and the devices have good consistency. Figure 2d shows the cumulative probability of resistance for the device’s 30 memory cells. The coefficient of variation in the resistance of the device HRS (R_HRS_) is 1.31, and the coefficient of variation in the resistance of the device LRS (R_LRS_) is 1.16, so the devices prepared in this paper have good reliability.

We conducted a device-to-device variability study to further confirm the reliability of the electrical characteristics. Specifically, 10 different devices were fabricated, and one cell was randomly selected from each device to measure its I–V characteristics. The results are presented in the Supporting Information (Figure S1), showing consistent I–V curves across all devices. Additionally, the HRS and LRS resistances of these devices were statistically analyzed, and the resistance distributions are shown in the Supporting Information (Figure S2). These results indicate good uniformity and reliability among devices, further supporting the robustness of the memristor performance.

The double logarithmic I–V characteristics under positive bias are shown in Figure 2e. In the high-resistance state (HRS), the slope of the fitted line is close to 1, indicating Ohmic conduction. As the voltage increases, the slope approaches 2, corresponding to a space-charge-limited current (SCLC) mechanism. After the SET voltage is reached, the device switches to the low-resistance state (LRS), where the I–V curve again shows a near-linear relationship, suggesting Ohmic behavior due to the formation of conductive filaments.

Figure 2f illustrates the schematic switching mechanism of the AuNP-based gelatin memory device. Due to the molecular structure of gelatin, which contains oxygen-bearing functional groups such as hydroxyl and carbonyl, oxygen vacancies can be naturally generated under an external electric field, serving as the initial defect sources for filament formation. In the initial high-resistance state (HRS), the Au nanoparticles (Au NPs) and oxygen ions are uniformly dispersed throughout the gelatin-based active layer. Due to the nanoscale dimensions of Au NPs, a significant Coulomb blockade effect exists, preventing electrons from hopping freely between nanoparticles, thus hindering charge transport and maintaining the device in HRS. When a negative voltage is applied to the top electrode, oxygen ions migrate downward toward the bottom electrode, leaving behind oxygen vacancies. As the voltage increases to the V_SET threshold, these oxygen vacancies align and form conductive filaments connecting the top and bottom electrodes. This results in a sudden current increase and switches the device to a low-resistance state (LRS). Subsequently, when a positive bias is applied, the previously migrated oxygen ions return and gradually fill the oxygen vacancies along the conductive paths. Once the voltage reaches the V__RESET_ point, the conductive filament ruptures due to reoxidation, breaking the path and restoring the HRS. This switching behavior supports a filamentary model dominated by oxygen vacancy dynamics and further enhanced by interfacial effects induced by embedded Au NPs. The presence of the Coulomb blockade effect in Au NPs contributes to higher resistance contrast, thereby improving the switching current ratio. Furthermore, to verify the non-metallic nature of the conductive filaments, we conducted temperature-dependent I–V measurements under the LRS condition. As shown in Figure S4, the current decreases with increasing temperature, exhibiting a negative temperature coefficient (NTC) behavior. This observation strongly supports the non-metallic and oxygen-vacancy-dominated nature of the conductive filaments.

**Figure 3:**
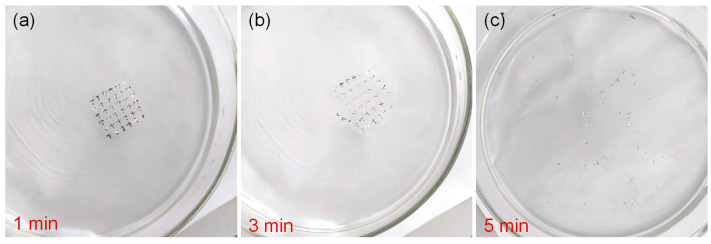
Water-soluble transient process of the Al/gelatin:Au NPs/Al/gelatin device. The process of device immersion in deionized water for (**a**) 1 min, (**b**) 3 min, and (**c**) 5 min.

Another attractive feature of Al/gelatin:Au NPs/Al/gelatin devices in data security is that gelatin is a water-soluble and physically transient material. The devices were soaked in deionized water at ambient temperature for 1, 3, and 5 min (Figure 3a–c). The surface topography of the device remained smooth and consistent at the beginning, but some irregular bumps appeared during immersion. After immersion in deionized water for 5 min, the device dissolves, which indicates the water solubility of the device and provides a broad prospect for the study of physical transient electronics.

**Figure 4:**
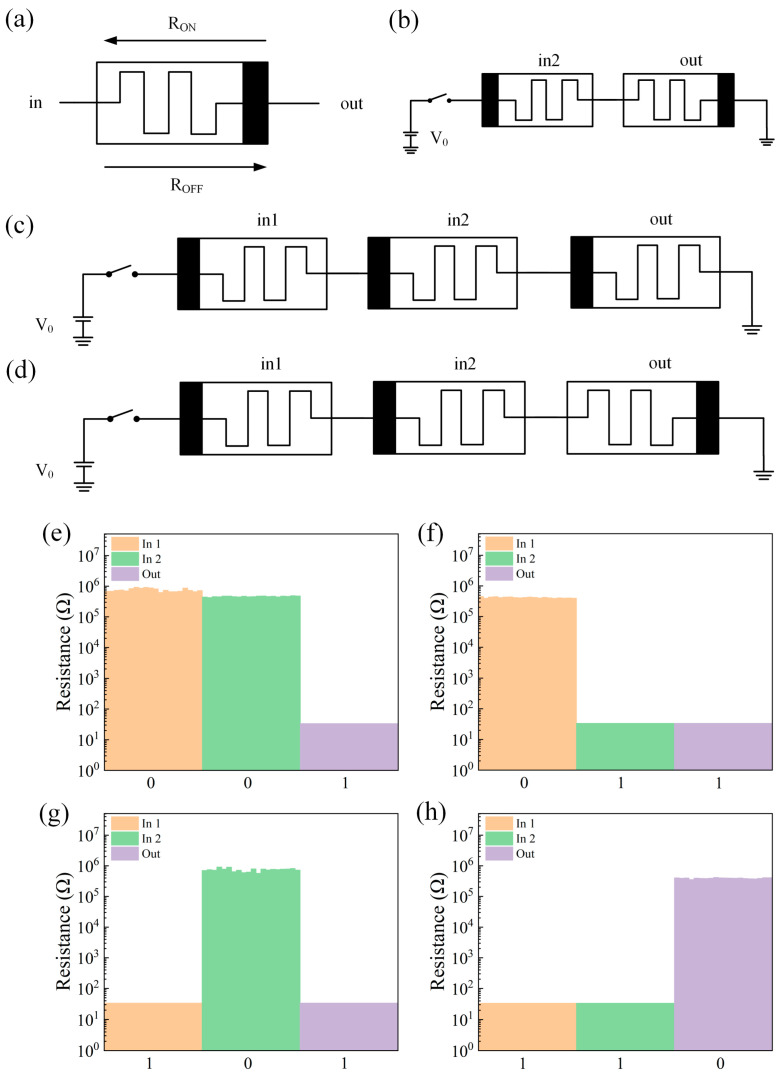
(**a**) The resistance change characteristics of the memristor. (**b**) NOT gate circuit schematic. (**c**) AND gate circuit schematic. (**d**) Schematic diagram of the NAND gate circuit. (**e**–**h**) Test results of the NAND gate logic circuit with different inputs (00,01,10,11).

The memristor logic gate in this paper is constructed by using MAGIC logic, and the resistance change characteristics of the memristor are specified as follows. As shown in Figure 4a, the polar end of the memristor is represented by black. When the current flows from the input end to the output end and the voltage exceeds the threshold voltage V_SET_, the device switches from the HRS to LRS, and the resistance value of the device decreases. When the voltage from the output terminal to the input terminal exceeds the threshold voltage V_RESET_, the state of the device is switched from LRS to HRS, where HRS is regarded as a logic 0 and LRS is regarded as a logic 1. The polar end is regarded as the bottom electrode of the device, and the other end is regarded as the top electrode of the device according to the switching characteristics of the device. The schematic diagram of the NAND gate based on MAGIC logic is shown in Figure 4d, which is changed on the basis of the NOT gate circuit of Figure 4b and the AND gate circuit of Figure 4c, and the input memristor is connected to the nonpolar end of the output memristor. First, switch the memristors in1 and in2 to the appropriate resistance state. According to Kvatinsky’s method [33], a voltage V0 is applied, and then the resistance state of the output memristor is read. The test results are shown in Figure 4e–h. The circuit realizes the logical function of NAND.

**Figure 5:**
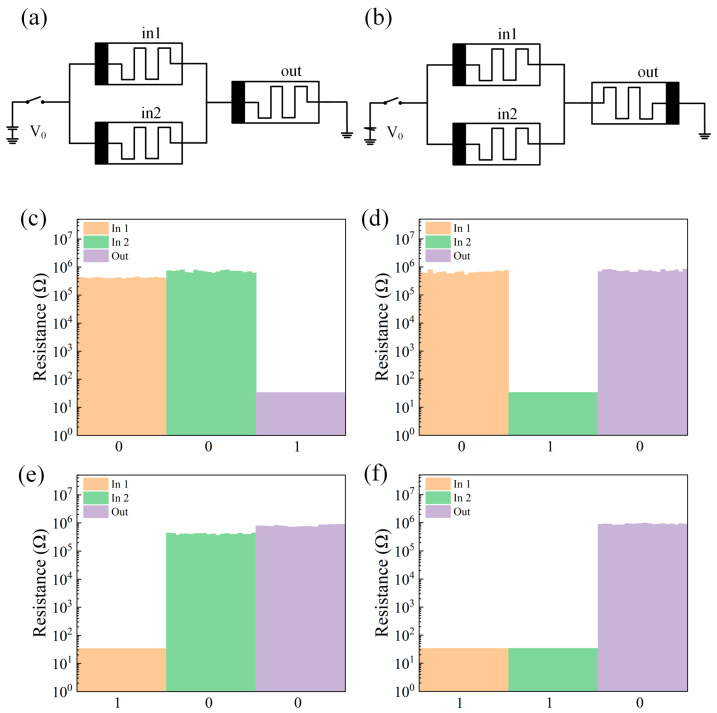
(**a**) OR gate circuit schematic. (**b**) NOR circuit schematic. (**c**–**f**). Test results of the NOR gate logic circuit with different inputs (00,01,10,11).

The circuit schematic diagrams of the OR gate and the NOR gate are shown in Figure 5a,b, and the test results are shown in Figure 5c–f. From the test results, it can be seen that the circuit realizes the logic function of NOR.

## 3. Conclusions

In conclusion, we fabricated a gelatin resistive memory based on Au NPs doped with the advantages of a simple process, biocompatibility, and transparency. Gelatin is doped with Au NPs. Due to the Coulomb blocking effect of Au NPs, the switching current of the device is relatively high, and the consistency is good. In addition, the device prepared on the gelatin substrate has physical transient properties. The biomemristor can be applied to the construction of MAGIC logic circuits, and a NAND and NOR logic gate circuit has been successfully constructed, which provides an effective way to research low-cost, green, environmentally friendly, and biocompatible electronic devices with logic functions. It is worth noting that although the presented device exhibits excellent water solubility, making it promising for transient electronics and disposable biosensors, its operational stability under harsh environmental conditions—such as humidity, high temperature, or dust—requires further improvement. Future studies may explore approaches such as gelatin matrix modification or protective encapsulation strategies to enhance environmental tolerance while maintaining the transient functionality.

## 4. Experimental Section

First, ITO/glass substrates were sequentially ultrasonically cleaned in acetone, ethanol, and deionized water for 10 min each, followed by natural air drying, and then heated at 80 °C for 10 min to remove residual moisture. Then, the Al bottom electrode was deposited onto the gelatin substrate by vacuum evaporation. The Au nanoparticle (NP) solution was synthesized as follows: 0.01 g of chloroauric acid was dissolved in 100 mL of deionized water and brought to a boil. Under continuous stirring at 600 rpm, 1 mL of 1 wt% trisodium citrate aqueous solution was added. The solution was maintained at boiling for approximately 20 min until it turned ruby red, indicating the formation of Au NPs. The solution was then naturally cooled to room temperature. Next, 0.5 mL of gelatin solution was added to 7.5 mL of the as-prepared Au NP solution, and the mixture was ultrasonicated for 5 min to obtain the gelatin:Au NP composite solution. This composite solution was drop-cast onto the gelatin substrate pre-patterned with Al bottom electrodes and dried at 100 °C for 10 min to form the active layer. Finally, the Al top electrode was deposited on the composite film by vacuum evaporation.

The electrical characteristics of the device were tested using a Keithley 4200 semiconductor parameter analyzer (Keithley, Solon, OH, USA) at ambient temperature and pressure. The morphology of Au NPs was observed using transmission electron microscopy (TEM, JEM-2100, JEOL, Tokyo, Japan).

## Figures and Tables

**Table 1:** Comparison of bio-memristor performance and functions.

Device Structure	Switching Current Ratio	Retention Time (s)	Functions	References
Al/gelatin:Au NPs/ITO	~7.68 × 10^6^	10^4^	NAND and NOR	This paper
Al/tussah hemolymph/ITO	~10^2^	10^4^	AND and OR	[27]
Ag/mugwort:PVDF/ITO	~3.6	10^5^	AND/OR/NAND/NOR/XOR	[28]
Ag/silk nanofibrils/ITO	10^2^	10^5^	AND and OR	[29]
Au/silk:AgNO_3_/Ag	3 × 10^6^	10^3^	short-term plasticity and paired-pulse facilitation	[30]
Ag/sericin/W	100	/	spiking rate-dependent plasticity and spiking time-dependent plasticity	[31]

## Data Availability

The datasets used and/or analyzed during the current study are available from the corresponding author on reasonable request.

## Supplementary Materials

The following supporting information can be downloaded at https://www.mdpi.com/article/10.3390/gels11060428/s1, The Supporting Information includes detailed electrical characterization results for 20 independently fabricated Al/gelatin:Au NPs/Al memristor devices, confirming device-to-device uniformity and reliability (Figures S1 and S2). Figure S1 presents the I–V characteristics of representative cells from each device, demonstrating consistent resistive switching behavior. Figure S2 provides a statistical analysis of the high-resistance state (HRS) and low-resistance state (LRS), showing narrow resistance distributions across all devices. Figure S3 shows the cross-sectional transmission electron microscopy (TEM) image of the Al/gelatin:Au NPs/Al device. The image clearly reveals the multilayer structure consisting of the top electrode, dielectric layer, and bottom electrode, confirming the structural integrity and thickness design of the device layers. Figure S4 presents the temperature dependence of the current in the low-resistance state (LRS). The results demonstrate that the current decreases with increasing temperature, indicating a negative temperature coefficient (NTC) behavior. This observation supports the non-metallic, oxygen-vacancy-based filament conduction mechanism proposed in the main manuscript.

## References

[1] Kurshan E., Li H., Seok M., Xie Y. (2020). A Case for 3D Integrated System Design for Neuromorphic Computing and AI Applications. Int. J. Semant. Comput..

[2] Wang L., Ding J., Pan L., Cao D., Ding X. (2019). Artificial Intelligence Facilitates Drug Design in the Big Data Era. Chemometr. Intell. Lab..

[3] Lu Z.X., Qian P., Bi D., Ye Z.W., He X., Zhao Y.H., Su L., Li S.L., Zhu Z.L. (2021). Application of AI and IoT in Clinical Medicine: Summary and Challenges. Curr. Med. Sci..

[4] Jagatheesaperumal S.K., Rahouti M., Ahmad K., Al-Fuqaha A., Guizani M. (2021). The Duo of Artificial Intelligence and Big Data for Industry 4.0: Applications, Techniques, Challenges, and Future Research Directions. IEEE Internet Things.

[5] Tripathi N., Goshisht M.K., Sahu S.K., Arora C. (2021). Applications of Artificial Intelligence to Drug Design and Discovery in the Big Data Era: A Comprehensive Review. Mol. Divers..

[6] Sun Y., Wang S., Zeng S., Huang X., Zhou P. (2021). Versatile Logic and Nonvolatile Memory Based on a Van Der Waals Heterojunction. ACS Appl. Electron. Mater..

[7] Niu X., Tian B., Zhu Q., Dkhil B., Duan C. (2022). Ferroelectric Polymers for Neuromorphic Computing. Appl. Phys. Rev..

[8] Carrillo S.G.C., Lugnan A., Gemo E., Bienstman P., Pernice W.H., Bhaskaran H., Wright C.D. (2021). System-Level Simulation for Integrated Phase-Change Photonics. J. Light. Technol..

[9] Zhang Y., Yao D., Liu Y., Fang C., Wang S., Wang G., Huang Y., Yu X., Han G., Hao Y. (2021). All-Optical Synapse with Directional Coupler Structure Based on Phase Change Material. IEEE Photonics J..

[10] Liao K., Chen Y., Yu Z., Hu X., Wang X., Lu C., Lin H., Du Q., Hu J., Gong Q. (2021). All-Optical Computing Based on Convolutional Neural Networks. Opto-Electron. Adv..

[11] Wang S., Liu X., Zhou P. (2021). The Road for 2D Semiconductors in the Silicon Age. Adv. Mater..

[12] Coluccio A., Casale U., Guastamacchia A., Turvani G., Vacca M., Roch M.R., Zamboni M., Graziano M. (2021). Hybrid-SIMD: A Modular and Reconfigurable Approach to Beyond von Neumann Computing. IEEE Trans. Comput..

[13] Kundu S., Ganganaik P.B., Louis J., Chalamalasetty H., Rao B.P. (2022). Memristors Enabled Computing Correlation Parameter In-Memory System: A Potential Alternative to von Neumann Architecture. IEEE Trans. VLSI. Syst..

[14] He Z.Y., Wang T.Y., Chen L., Zhu H., Sun Q.Q., Ding S.J., Zhang D.W. (2019). Atomic Layer-Deposited HfAlOx-Based RRAM with Low Operating Voltage for Computing In-Memory Applications. Nanoscale Res. Lett..

[15] Sung S.H., Kim D.H., Kim T.J., Kang I.S., Lee K.J. (2019). Unconventional Inorganic—Based Memristive Devices for Advanced Intelligent Systems. Adv. Mater. Technol..

[16] Li Y., Wang Z., Midya R., Xia Q., Yang J.J. (2018). Review of Memristor Devices in Neuromorphic Computing: Materials Sciences and Device Challenges. J. Phys. D Appl. Phys..

[17] Sun K., Chen J., Yan X. (2021). The Future of Memristors: Materials Engineering and Neural Networks. Adv. Funct. Mater..

[18] Kang C.F., Kuo W.C., Bao W., Ho C.H., Huang C.W., Wu W.W., Chu Y.H., Juang J.Y., Tseng S.H., Hu L. (2015). Self-Formed Conductive Nanofilaments in (Bi, Mn) Ox for Ultralow-Power Memory Devices. Nano Energy.

[19] Bai W., Huang R., Cai Y., Tang Y., Zhang X., Wang Y. (2013). Record Low-Power Organic RRAM with Sub20-nA Reset Current. IEEE Electr. Device Lett..

[20] Ahn S.E., Lee M.J., Kang B.S., Lee D., Kim C.J., Kim D.S., Chung U.I. (2012). Investigation for Resistive Switching by Controlling Overflow Current in Resistance Change Nonvolatile Memory. IEEE Trans. Nanotechnol..

[21] Wu H., Wang X.H., Gao B., Deng N., Lu Z., Haukness B., Bronner G., Qian H. (2017). Resistive Random Access Memory for Future Information Processing System. Proc. IEEE.

[22] Trotti P., Oukassi S., Molas G., Bernard M., Aussenac F., Pillonnet G. (2021). In Memory Energy Application for Resistive Random Access Memory. Adv. Electron. Mater..

[23] Covi E., Wang W., Lin Y.-H., Farronato M., Ambrosi E., Ielmini D. (2021). Switching Dynamics of Ag-Based Filamentary Volatile Re-Sistive Switching Devices—Part I: Experimental Characterization. IEEE Trans. Electron Dev..

[24] Wang X.F., Tian H., Zhao H.M., Zhang T.Y., Mao W.Q., Qiao Y.C., Pang Y., Li Y.X., Yang Y., Ren T.L. (2018). Interface Engineering with MoS2–Pd Nanoparticles Hybrid Structure for a Low Voltage Resistive Switching Memory. Small.

[25] Shen Z., Zhao C., Qi Y., Xu W., Liu Y., Mitrovic I.Z., Yang L., Zhao C. (2020). Advances of RRAM Devices: Resistive Switching Mechanisms, Materials and Bionic Synaptic Application. Nanomaterials.

[26] Duan W., Wang J., Zhong X. (2018). Electrically Controlled Nonlinear Switching and Multilevel Storage Characteristics in WOx Film-Based Memory Cells. J. Phys. Chem. Solids.

[27] Wang L., Zhang Y., Wen D. (2021). Flexible Nonvolatile Bioresistive Random Access Memory with an Adjustable Memory Mode Capable of Realizing Logic Functions. Nanomaterials.

[28] Lin F., Cheng Y., Li Z., Wang C., Peng W., Cao Z., Gao K., Cui Y., Wang S., Lu Q. (2024). Data encryption/decryption and medical image reconstruction based on a sustainable biomemristor designed logic gate circuit. Mater. Today Bio.

[29] Zhang Y., Fan S., Niu Q., Fang H., Zhang Y.P. (2022). Intrinsically ionic conductive nanofibrils for ultrathin biomemristor with low operating voltage. Sci. China Mater..

[30] Zhao M., Wang S., Li D., Wang R., Li F.F., Wu M.Q., Liang K., Ren H.H., Zheng X.R., Guo C.C. (2022). Silk protein based volatile threshold switching memristors for neuromorphic computing. Adv. Electron. Mater..

[31] Rong H.H., Zhang M.C., Liang X., Liu C., Saadi M., Chen X.Y., Yao L., Zhang Y.R., He N., Hu E. (2023). Demonstration of electronic synapses using a sericin-based biomemristor. Appl. Phys. Express.

[32] Tungkavet T., Pattavarakorn D., Sirivat A. (2012). Biocompatible Gelatins (Ala-Gly-Pro-Arg-Gly-Glu-4Hyp-Gly-Pro-) and Electro-Mechanical Properties: Effects of Temperature and Electric Field. J. Polym. Res..

[33] Kvatinsky S., Wald N., Satat G., Kolodny A., Weiser U.C., Friedman E.G. (2012). MRL—Memristor Ratioed Logic. Proceedings of the 2012 13th International Workshop on Cellular Nanoscale Networks and Their Applications.

