# Supplementary material for: Physically Transient Gelatin-Based Memristors of Buildable Logic Gates
Source: Gels. 2025 Jun 3;11(6):428. doi: 10.3390/gels11060428 (PMC12192105; doi:10.3390/gels11060428)
Supplement: Supplementary file 1 [file gels-11-00428-s001.zip › gels-3341229-supplementary.pdf]

Supporting Information

Physically transient gelatin-based memristors of buildable logic gates

Lu Wang *, Yuting Wang, Wenhao Li, Zhiqiang Gao, Yutong Han and Dianzhong Wen

School of Electronic Engineering, Heilongjiang University, Harbin, 150080, China;

*Correspondence: wanglu@hlju.edu.cn

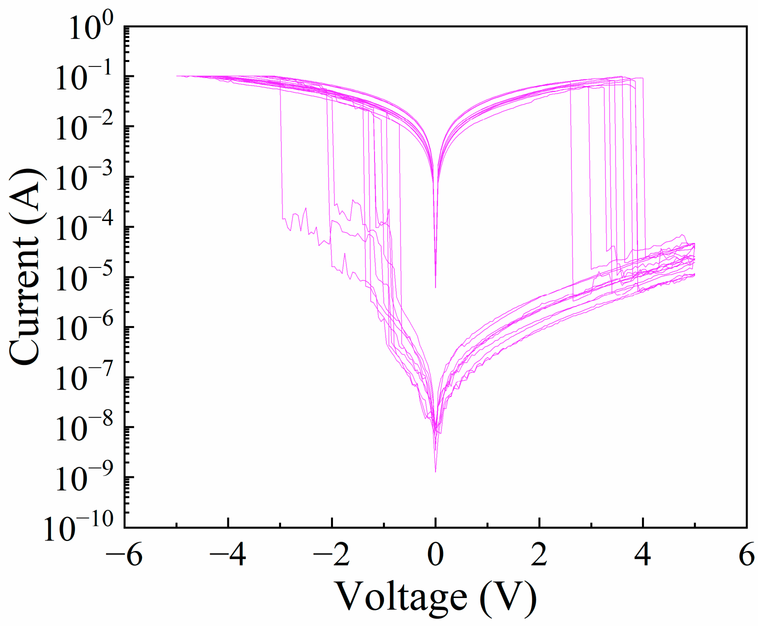

Figure S1. The I-V characteristics of 10 different devices, each with one randomly selected cell. The results show consistent electrical behavior across devices, confirming the uniformity and reliability of the memristor fabrication process. To simplify the measurement process and ensure consistency, a limiting current of 100 mA was used for both the negative and positive bias regions.

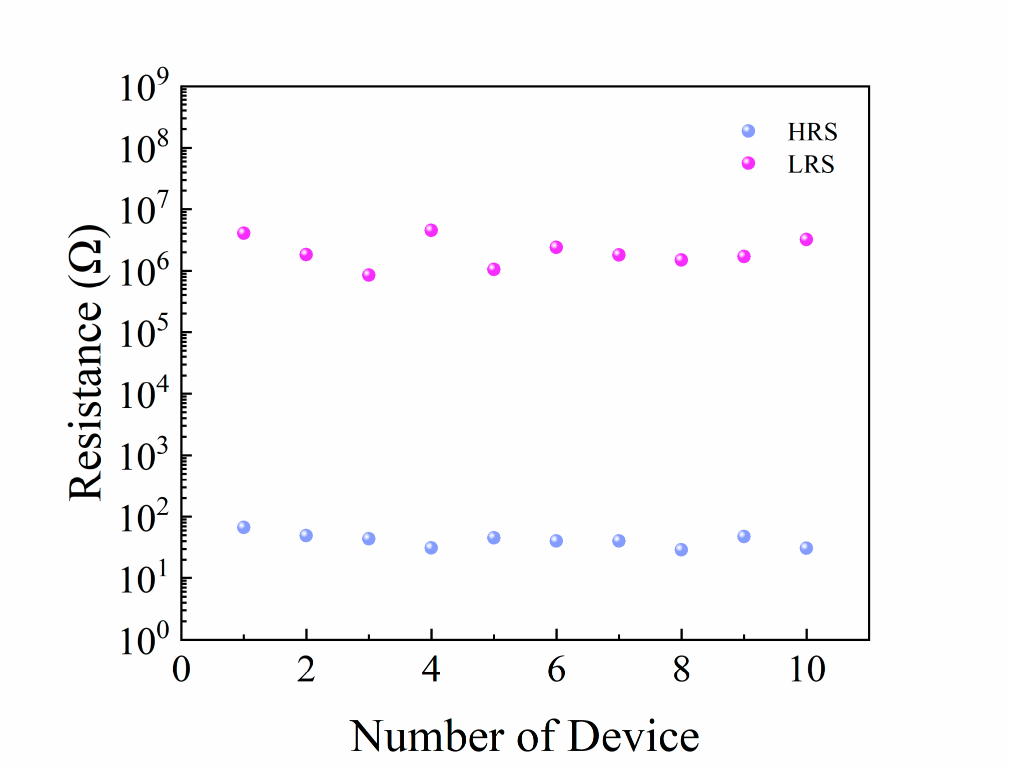

Figure S2. Statistical analysis of HRS and LRS resistances for the 10 devices. The resistance distributions indicate good consistency among devices.

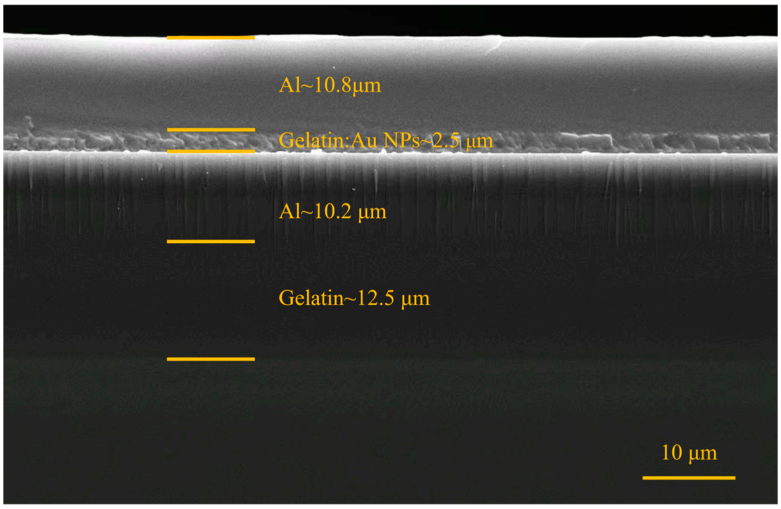

Figure S3. Cross-sectional TEM image of the Al/gelatin:Au NPs/Al device structure. The layer thicknesses are approximately: bottom gelatin layer ~12.5 μm, followed by the Al electrode layer ~10.2 μm, the gelatin:Au NPs composite layer ~2.5 μm, and the top Al layer ~10.8 μm. The scale bar is 10 μm.

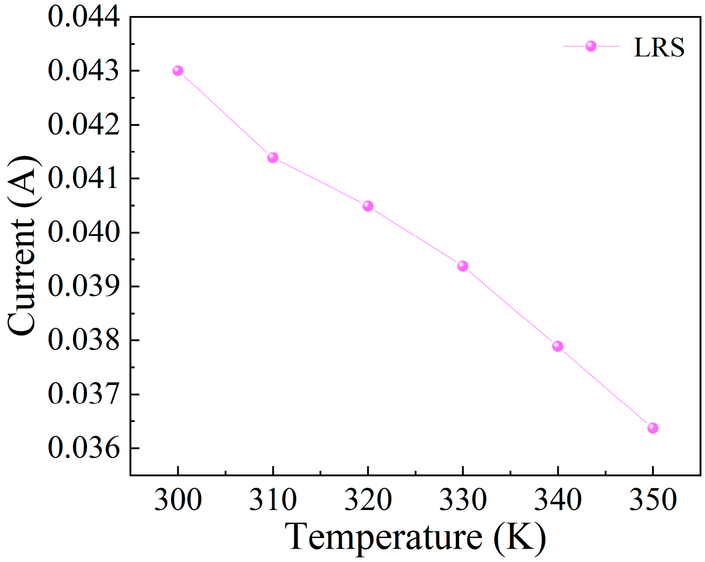

Fig. S4. Temperature dependence of the current in the low resistance state (LRS) of the Al/gelatin:Au NPs/Al/gelatin device, showing a negative temperature coefficient behavior.
